# Establishment of an HLA-typed cohort of 1000 healthy blood donors and use of a dendritic cell priming assay to prime naive T cells to structurally divergent drugs

**DOI:** 10.1186/2045-7022-4-S3-P112

**Published:** 2014-07-18

**Authors:** Lee Faulkner, Sally Wood, Diane Van Eker, Ana Alfirevic, Munir Pirmohamed, Dean Naisbitt, Kevin Park

**Affiliations:** 1 University of Liverpool, MRC Centre for Drug Safety Science, UK

## Background

The mechanisms involved in the etiology of adverse drug reactions (ADRs) are complex. A small proportion of ADRs are due to drug hypersensitivity where the immune response causes an unexpected and severe clinical reaction. The mechanisms of drug hypersensitivity are not yet fully understood, but genome wide screens have identified an association with HLA alleles and certain drugs. Furthermore, functional studies have identified drug-responsive T-cells in blood and tissues of hypersensitive patients. Thus, the frequency and severity of a reaction is a function of the chemistry of the drug, the biology of the immune cell and the genotype of the individual.

## Method

We sought to establish a biobank of lymphocytes isolated from HLA-typed volunteers to investigate how drugs and their metabolites become antigens and interact with the HLA molecules and T cells to generate an immune response. We have recruited 900 volunteers so far and 400 of these have been HLA typed.

## Results

The typed cohort is representative of the North-West population and consists of 64% female and 36% male volunteers with a mean age of 29 years (+/-10 years, range 18-60 years). The ethnicity is primarily Caucasian (84%); however, a special effort was made to include other ethnicities (Asian Indian 6%, Chinese 4%, Black 1%, Other 5%) in order to increase the HLA allelic diversity within the cohort. All major Caucasian haplotypes are represented. Using a DC priming assay we have investigated whether we could prime naïve T cells to various drugs. Nitroso sulfamethoxazole was included in all assays as a positive control and drug-specific proliferative responses were generated from 35 out of 38 donors. Strong responses were also generated to carbamazepine in two HLA-B*1502 donors (n=2), to flucloxacillin in one HLA-B*5701 donor (n=5), to pipericillin in 5 donors (n=10) and to Bandrowski's base in all donors (n=7). In contrast, no positive responses were detected to ximelagatran in four HLA-DRB1*0701 donors and to lumiracoxib in four HLA-DRB1*1501 donors. However, drug-specific T cell clones could still be isolated from 3 donors when only weak responses to flucloxacillin were detected.

## Conclusion

This work shows that drug specific responses can be generated from naïve T cells and suggests that not all hypersensitivity reactions are due to cross-reactivity with existing memory cells.

